# Evaluation of Low-Level Laser Therapy in TMD Patients

**DOI:** 10.1155/2015/424213

**Published:** 2015-10-26

**Authors:** Simel Ayyildiz, Faruk Emir, Cem Sahin

**Affiliations:** ^1^Department of Prosthodontics, Center of Dental Sciences, Gulhane Military Medical Academy, Etlik, 06018 Ankara, Turkey; ^2^School of Dental Technology, Hacettepe University, 06100 Ankara, Turkey

## Abstract

Light amplification by stimulated emission of radiation (laser) is one of the most recent treatment modalities in dentistry. Low-level laser therapy (LLLT) is suggested to have biostimulating and analgesic effects through direct irradiation without causing thermal response. There are few studies that have investigated the efficacy of laser therapy in temporomandibular disorders (TMD), especially in reduced mouth opening. The case report here evaluates performance of LLLT with a diode laser for temporomandibular clicking and postoperative findings were evaluated in two cases of TMD patients. First patient had a history of limited mouth opening and pain in temporomandibular joint (TMJ) region since nine months. Second patient's main complaint was his restricted mouth opening, which was progressed in one year. LLLT was performed with a 685 nm red probed diode laser that has an energy density of 6.2 J/cm^2^, three times a week for one month, and application time was 30 seconds (685 nm, 25 mW, 30 s, 0.02 Hz, and 6.2 J/cm^2^) (BTL-2000, Portative Laser Therapy Device). The treatment protocol was decided according to the literature. One year later patients were evaluated and there were no changes. This application suggested that LLLT is an appropriate treatment for TMD related pain and limited mouth opening and should be considered as an alternative to other methods.

## 1. Introduction

TMD are about a variety of clinical problems that take their origin from TMJ, masticatory muscles, and surrounding tissues [1]. The cause of pain in the orofacial region that does not take its origin from dental arches is mostly the TMD [2]. In the community 40% to 75% of healthy individuals point out at least one sign and 33% observed at least one symptom of TMD [3]. The most affected anatomical structures of the TMJ from these diseases are posterior attachments, collateral ligaments, and periarticular tissues (capsule, synovium, and temporomandibular joint ligaments).

Distinction based on the definition of TMD is generally limited to muscular and systemic originated diseases of the joint [4]. However, relationship between these two groups is often closely related to each other. Thus, accurate diagnosis of these diseases is the most important point. In clinic, physical therapy (conservative treatment) and surgical procedures (invasive treatment) are used to treat TMD that based on intra-articular pathologies [3]. Anti-inflammatory drugs, soft diets, notifications, occlusal splints, and low-level laser treatments are the most widely used conservative treatments. Surgical procedures mainly include arthrocentesis, intra-articular injections, arthroscopy, and open joint surgery. These techniques are used alone or can be the combination of two methods.

In recent years, laser light is used successfully in dentistry in clinic for the treatment of soft tissue diseases [5–7], dentinal hypersensitivity [7–10], bone regeneration [7, 11], and musculoskeletal pain [7, 12]. Among conservative treatment procedures LLLT has been used in TMD patients to improve function and reduce symptoms [3].

LLLT, which is classified as a soft laser, has low-level energy output and does not affect skin temperature. LLLT's main effect is based on the mechanism of light absorption. The wavelength of this soft laser is ranging between 630 and 1300 nm [3]. In spite of the unclear operating mechanism of LLLT, it stimulates tissues and has an analgesic, anti-inflammatory effect via direct irradiation [3]. The stimulation of LLLT affects the cellular respiratory chain in the mitochondria that induces increased vascularization and fibroblast formation. LLLT not only affects the blood microcirculation but also increases ATP production. LLLT gives an increase of lymphatic flow that reduces edema and causes decrease of prostaglandin E2 and cyclooxygenase-2 levels [3]. Under the surface of the skin at 1 cm depth the intensity of a laser is reduced to 10% of its value. Therefore a laser with a power density of 100 mW/cm^2^ at the surface of the skin will be 10 mW/cm^2^ at 1 cm below and at 2 cm below will be 1 mW/cm^2^ [13]. This data shows that LLLT is safe enough for using in TMD region, but its effect in treatment procedure should be well examined clinically.

Therefore the aim of this study was to evaluate the effect of the diode laser on TMD patients for the control of pain and limited mouth opening.

## 2. Case Report

**Figure 1:**
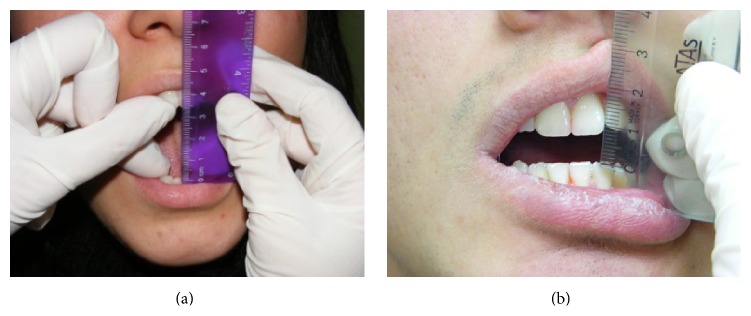
Mouth opening of both patients before treatment.

**Figure 2:**
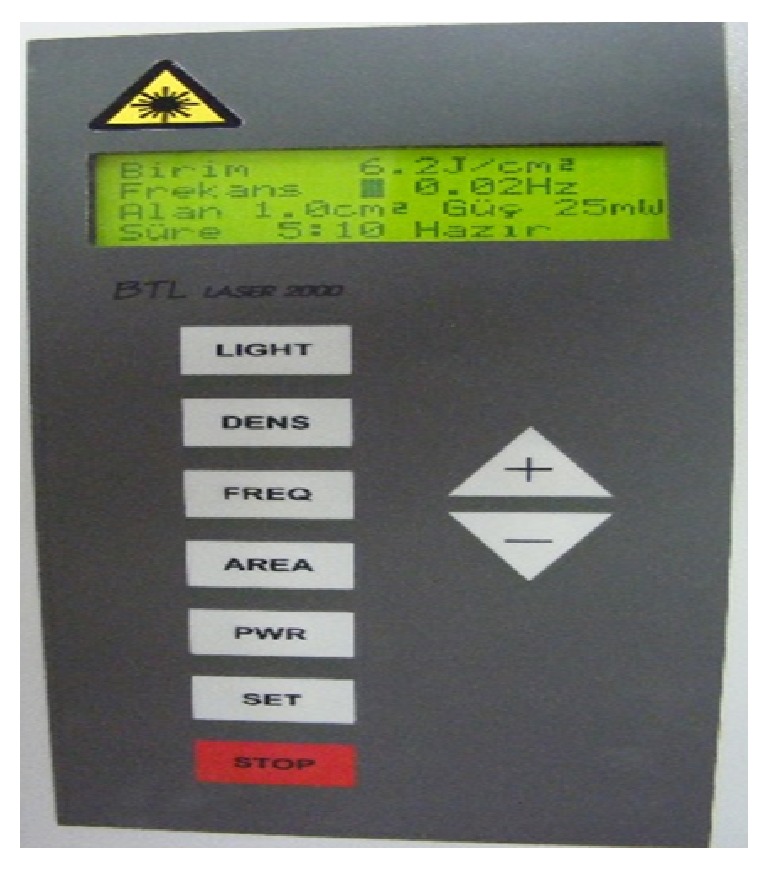
Laser device.

A 25-year-old female patient had referred to our department with complaints of limited mouth opening and pain in TMJ region continuing for nine months. Medical and dental histories were recorded. The medical history of the patient revealed that she has any systemic disease that was defined before. In dental examination there was not any teeth loss but limited mouth opening was determined. For this reason, magnetic resonance imaging (MRI) was taken. According to the report of MRI there was anterior disc dislocation without reduction in both sides. In questioning there was no history of trauma but she was a student and was preparing for an important exam, so she had nocturnal and diurnal tooth grinding. She had visited another dental clinic with the same complaints; therapeutic therapy and an occlusal splint have been advised. Although she had used the splint, myorelaxant, and analgesic pills for 3 months there was not any change in limited mouth opening (Figure 1). The clinical examination revealed bilateral TMJ pain during opening and lateral movements. The maximum mouth opening (MMO) was 34 mm, left excursions (LE) and right excursions (RE) were 5 mm separately, and the patient was feeling pain at these limits. The muscle examination revealed no pain or tenderness. The patient was instructed about the LLLT and a free informed consent form was obtained from her. LLLT was performed with a 685 nm red probed diode laser that has an energy density of 6.2 J/cm^2^, three times a week for one month, and application time was 30 seconds (685 nm, 25 mW, 30 s, 0.02 Hz, and 6.2 J/cm^2^) (BTL-2000, Portative Laser Therapy Device) (Figure 2). The treatment protocol was decided according to the literature [6–9].

**Figure 3:**
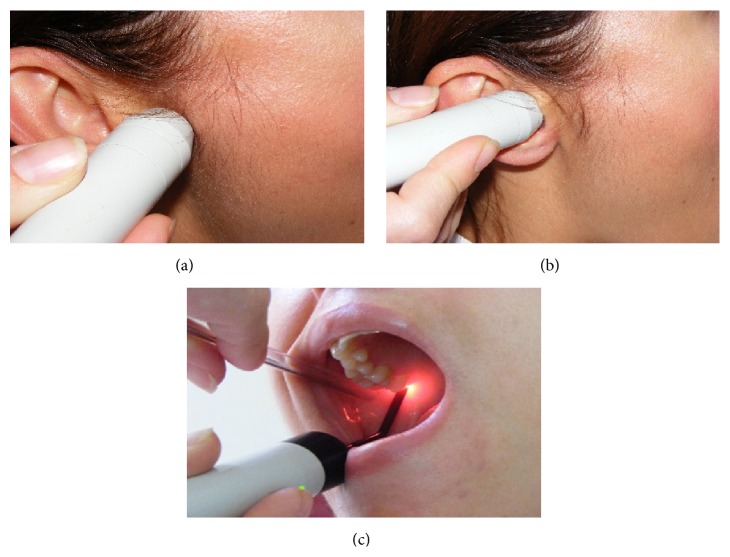
Laser treatment regions.

**Figure 4:**
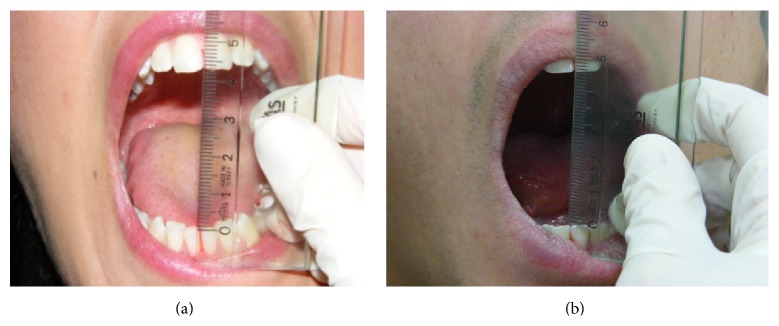
Mouth opening of both patients after treatment.

**Figure 5:**
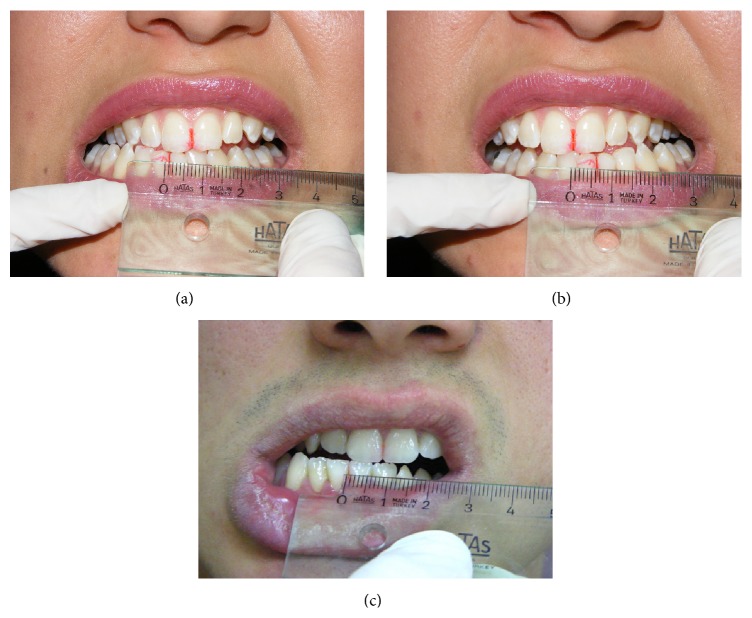
Lateral movement of both patients after treatment.

Laser beam was applied at three points in each TMJ: (a) the posterior aspect of the joint in maximum opening to treat the posterior articular branches of the auriculotemporal nerve and posterior discal attachment region by applying the beam from the anterior of the external auditory channel and (b) same region in maximum opening from inside the external auditory channel (c) to the inferior branches of the medial pterygoid muscle with the fine fiber optic probe of the device from inside the mouth through the posterior of the tuber maxilla (Figure 3). In every session, the patient marked the VAS scale (0–10 cm) before and after the treatment. Also in every session, maximum mouth opening, left and right lateral excursion of the patient, was recorded. At the end of the treatment an occlusal splint was fabricated as a night guard and the patient was informed about the use of this splint. Also the patient was evaluated immediately after the application and at the follow-up appointments after 15 days, 1, 3, and 6 months of the end of the treatment, to investigate effectiveness and cumulative effects. The mouth opening of the patient was increased gradually during the sessions. At the end of the treatment MMO was 45 mm (Figure 4), RE and LE were 8 and 6 mm, respectively (Figure 5), and she was painless during these limits of movement. Six months later there was a little relapse at clinical evaluation; MMO was 42 mm and RE and LE were 6 mm, but any pain was recorded during evaluation or function.

The second case was 18-year-old male patient, who was a student in Military School and also he was a regular kick boxer. The main complaint was his restricted mouth opening that was progressed in one year due to the impact taken to the mandible in an exercise (Figure 1). In the extraoral inspection of the TMJ, the masseter and temporal muscle region were palpated normally; there was any hypertonicity or hypersensitivity. But the posterior region of the condyle that was palpated from the meatus acusticus externus was hypersensitive during opening. The ligament of the anterior temporal muscle that was passing through the ramus was also sensitive during intraoral examination. It was recorded from the intraoral examination that there were not any caries or extracted tooth and maxillomandibular relation was Angle Class I. The interincisal midline was coincided with the facial midline, the MMO was 9 mm, and both LE and RE were 1 mm. The patient was instructed about the LLLT and a free informed consent form was obtained from him. The same curing method of the first patient was applied to the patient with the same protocol. At the end of the treatment an occlusal splint was fabricated as a night guard and the patient was informed about the use of this splint. Also, the patient was evaluated immediately after the application and at the follow-up appointments after 15 days, 1, 3, 6, and 12 months of the end of the treatment, to investigate effectiveness and cumulative effects. The MMO of the patient was increased gradually during the sessions. After the last application of the treatment MMO was increased to 45 mm (Figure 4), RE and LE were also increased to 8 mm (Figure 5), and he was painless during these limits of movement. There were no changes in the MMO in 1-year follow-up.

## 3. Discussion

LLLT utilizes electromagnetic radiation at a particular wavelength and contributes management of pain, impaired wound healing, and inflammations [14, 15]. Also, LLLT is usually used clinically for the treatment of TMJ pain. The therapeutic doses and output power are less than 35 J/cm^2^ and 500 mW, respectively [16].

In our study red probe diode laser (685 nm, 25 mW, 30 s, 0.02 Hz, and 6.2 J/cm^2^) was used in TMJ region at three points, including one point for intraoral and two different points for extraoral regions. These applications were made in three times a week for one month for each patient.

In some studies [17–19] the efficiency of 632 nm wavelength for laser treatments was found better than short wavelength lasers and the former penetrates musculoskeletal tissues better [19, 20]. Additionally some authors [19] reported that 632 nm lasers were more effective in pain reduction than 820 nm. Therefore the wavelength of 685 nm, which was used in this study, can be regarded as effective.

In literature, many authors report different energy densities for the treatment with low-level lasers. This value ranges from 1 to 35 J/cm^2^ [14, 15, 19, 21–30]. The energy density of the portable laser therapy device that was used in the treatment of two patients in this case report was 6.2 J/cm^2^; however this value was in the energy density limits of laser devices that were used by other researchers [21–30].

In the literature there is still no consensus on frequency of low-level lasers and number of sessions of laser applications. On frequency and number of application sessions, some authors [27, 31–33] discussed eight sessions with application twice per week. On the other hand some authors [26, 28, 33] found that six sessions with application of twice per week would be proper. And also some authors agreed in the number of ten sessions but in terms of frequency each one used different values [33]. The treatment protocol for two patients in this study was three times a week for one month. The aim of this protocol was to protect the obtained mouth opening of the patient after each session. Thus, the effectiveness of treatment and patient motivation were enhanced.

In the literature the application points of LLLT are also varying. In general, the application sites are through the overlying skin of the masseter, temporalis, and pterygoid muscles. Almost no intraoral application was observed. In this study laser device's special probe let us intraoral application. The intraoral application region was rear of the tuber, during mouth opening, which was located on the closest point to TMJ. Thus LLLT was performed on not only outside of the TMJ but also the closest region inside the mouth. The aim of this application protocol was to reach most near parts of the TMJ.

In literature, discussions on the effectiveness of LLLT are still continuing. Many studies report that the use of LLLT in TMD could be effective while the others report that its effectiveness is not fully proven. Particularly, Emshoff et al. [19] and De Abreu Venancio et al. [26] reported that there was no relief in TMJ pain after the application of LLLT. Also, Petrucci et al. [34] reported that LLLT is inadequate in reducing chronic TMJ pain. In literature there some studies that support that sentiment [34–36]. However, many studies reported that LLLT application is an effective therapy and can be used in the TMD patients [14, 21–25, 27–30]. The studies of Mazzetto et al. [27], Çetiner et al. [29], and Venezian et al. [32] reported that patients were followed up to 30 days after the last sessions of laser application. Çetiner et al. [29] and Venezian et al. [32] reported that the reduction in pain continued to be statistically significant in this period. Despite these results Mazzetto et al. [27] reported that the least sensitivity to palpation was seen in the last laser application session [27]. Lassemi et al. [37] followed up the patients for over 2 years and observed relevant results in pain reduction and clicking.

Thus, in our clinical study a noticeable relief was observed after the LLLT treatment in both patients and this condition was continued through 1-year follow-up. There was no reduction in MMO or lateral movements either. Furthermore pain and tenderness to palpation were not observed clinically.

Nonstandardized results could be obtained in consequence of using several methods. These results were thought to occur due to the factors like types, frequencies, and time periods of low-level laser radiation in different groups of patients [23]. For revealing the positive effects of LLLT, an accurate diagnosis and sufficient application protocol should be the key factors [38, 39].

In the treatment of TMD, several therapies such as acupuncture, transcutaneous electrical nerve stimulation (TENS), massage, ultrasound, pharmacotherapy, occlusal splints, and psychological treatments were used as alternative methods [33]. However, LLLT is an easily tolerated, noninvasive, and nonpharmaceutical treatment. It is a timesaving method for both clinician and patient and also has a rapid effect that can be felt by the patient after the application [19].

## 4. Conclusion

This application suggested that LLLT is an appropriate treatment for TMD related pain and limited mouth opening and should be considered as an alternative to other methods.
